# Monthly Summary

**Published:** 1878-08

**Authors:** 


### MONTHLY SUMMARY.

---

*Rules for Chloroform Administration.*—Prof. Syme had given chloroform, he said, for many years without an accident. He attributed his success to two causes. First, he always used good chloroform, and second, he always gave plenty of it.

*Antiseptics have no Action on the Blood.*—Dr. Laborde has lately published his experience with antiseptics, of which the substance is as follows:

A dog having been made septicæmic by the direct introduction of septic blood into a vein, the femoral artery is laid bare, and its central end is placed in direct communication with the separated dital end of the femoral artery of a healthy dog. The second dog, which receives the septicæmic blood in this manner, becomes itself septicæmic. By successive "generations" produced by this operative procedure a blood is obtained containing the septicæmic poison in a high degree of intensity. In regard to the presence of organisms in this morbigenic fluid, Dr. Laborde remarks that "the blood of a dog infected by arterial communication has never, when attentively examined at different periods of the disease, exhibited the presence, in appreciable quantity, of microcytes—bacteria, vibrios, granular matter, etc. —not even in cases in which the blood primarily inoculated contained microza in more or less large numbers; so that we find ourselves in presence of a disease which can be transmitted indefinitely without the intervention of inferior organsims." In contradiction to the statements of Binz, the author found that quinine, even when injected in the highest doses compatible with life, neither acted as a preventive to the development of the disease, nor arrested it when it was produced. Similar negative results were found with chromic acid, carbolic acid, bichromate of potash, and permanganate of potash.—*Druggists Circular.*

---

*Dangers from Horse back Exercise.*—Dr. Montzel, in the *Annales Medico-psycholoqiques*, gives a description of the so-called "malady of the Scythians," a race who may be said to live on horseback.

The essential features of this disease are loss of virility at an early age, and an alteration in the skin of the face and body. The features resemble those of a woman, while the whole habits of life are changed, gradually approximating those of the opposite sex. The skin becomes wrinkled, the beard disappears, the body loses greatly in strength, and the patient often assumes the costume of a woman. The men among whom these patients are found spend a great part of their lives on horseback, and most authors agree in regarding this as the chief cause of the disease, giving rise to spermatorrhœa and habits of masturbation.

In this country, we have known young men who had to avoid horseback exercise, on account of its inducing in them the same results.—*Med. and Surg. Reporter.*

*Opium vs. Coffee.*—M. Richet says, in the *Revue des Deux Mondes*:—

Opium has its antidote; just as we can produce sleep, so, too, can we produce sleeplessness, by the employment of a mind poison, whose effects are diametrically opposite to those of the other. The antedote of opium is coffee. One hundred years ago coffee was almost unknown, but now there is hardly another beverage that is so widely distributed. Every one has it in his power to judge of the effects of coffee. For some persons it is a stimulus necessary for the performance of intellectual work. In others it produces a painful state of insomnia; taken even in weak doses it causes restlessness and anxiety, a sort of feverish activity altogether different from the indolent activity of opium. Under the action of opium the will seems to be lulled to sleep and the imagination runs riot. But, under the influence of coffee, the imagination is hardly stimulated at all, while there does appear to be excitation of the will. Did I not fear being suspected of having a theory to defend, I should say that the faculties of will and consciousness seem to be superexcited; there is, as it were, a constant strain on attention and memory, whereas, in the case of alcohol, hasheesh and opium, there is a relaxing of attention. Hence coffee produces a true intoxication that fatigues one far more than does the somnolent intoxication of opium, but it leads to the same result. In striving to do too much, the mind does less; under stimulation the will is impaired, and the perfect equilibrium of the mental faculties is disturbed as well by excess as by defect of will.

---

*Absorption of Tincture of Iodine by the Skin.*—Dr. L. Menager has experimented upon children with a solution of equal parts of tincture of iodine and glycerine, rubbed into the skin, and has arrived at the following conclusions. 1. Iodine in tincture, mixed with glycerine and applied to the external integument, is absorbed. 2. This absorbed iodine is invariably found in the secretion and in the urine. (Dr. M. tests for iodine by adding a little starch to the urine in a test-tube and then dropping a few drops of nitroso nitric acid into it. This gives a blue or violet color, according to the quantity of starch present.) 3. This application may give rise to certain symptoms, usually a variety of mild temporary albuminuria. 4. Dressings containing tincture of iodine may be employed as a means of introducing this medicine into the system when it cannot be taken by the stomach. 5. It must not be forgotten that when this absorption takes place in patients subject to nervoso-vacular erethism, aa in certain cases of phthisis, where these dressings are often practised, they may do more harm than good.—*Dublin Med. Press and Circ.*

*Thymol as an Antiseptic.—American Journal of Med. Sciences.*—Thymol is being used as a substitue for carbolic acid, in Lister's antiseptic dressings, by Prof. Volkmann at his clinic at Halle. The solution is prepared as follows: Thymol 1 part, alcohol 10 parts, glycerine 20 parts and water 1,000 parts. This solution has no corrosive action on the instruments immersed in it, in this respect being superior to carbolic acid, and far superior to salicylic acid.

When sprayed over the skin it produces a lively sense of burning, and a redness; otherwise it has no irritant qualities. Anæsthesia of the skin and desquamation of the epidermis do not follow its use, as they are liable to, under the use of carbolic acid. Nor does the thymol irritate the air passages if it is respired.

Antiseptic gauze, by this method, is made of bleached gauze 1,000 parts, spermaceti 500 parts, resin 50 parts and thymol 16 parts—spermaceti being substituted for the paraffin. The thymol gauze is applied directly to the wound—no protective being necessary because the thymol is non-irritant. Between the 7th and 8th external layers a piece of gutta-percha paper, which has been washed in the thymol solution, is placed, and the whole secured by a roller of the gauze. These dressings seldom need changing oftener than once in six or eight days, thus making a great saving over the carbolic dressings. The thymol gauze should be kept in stock wrapped in parchment paper, and should only be opened at the time it is needed.—*Detroit Lancet.*

---

*Iodoform as a Local Application.*—Chloroform is its best solvent when we wish to employ it in solution. It can be made into an ointment with lard or vaseline, and its odor disguised with some of the essential oils. As a powder it may be used alone or diluted with fuller's-earth, magnesia or tannin; the latter in some measure removes its disagreeable odor. Its action can, perhaps, be best shown, by stating its effects in the several affections in which he had used it. Venereal sores: It acts equally well in hard and soft chancres, no matter where located. It should not be applied to a sore when acutely inflamed. Cures are effected in rather less than half the time required for the ordinary local method. There is less risk of buboes, and the constitutional disturbance is not apt to be as great. It acts particularly well in cases where there is a disposition to slough. Buboes, syphilitic ulcerations, chronic ulcers, etc., he has found to speedily heal under its use when other means have entirely failed. It acts well as a parasiticide. In cases of ringworm of the scalp of long duration, speedy improvement ensued from its use in the form of ointment. Chloasma yields readily to it. In sycosis the results from its application were not encouraging, as it gave rise to undue irritation.—*Cottle, in British Med. Jour.*

*New Method of Plugging.*—Dr. Weil, of Munich, has employed and advocated the method of first extracting the tooth, filling it with amalgam or gold, and then replacing it. He states that the results are excellent, and the teeth can be freely used. He keeps the tooth out of the socket for one or two hours, as may be necessary, and yet the tooth ultimately is firmly fixed. He finds the method quite applicable to both bicuspids and molars. Since extraction can be performed under anæsthetics, many persons will prefer the new method to the old, provided the subsequent refixing does not involve more than complementary pain, and provided also the method is found as successful in other hands as in those of the inventor's.—*Med. and Surg. Reporter.*

---

*To Utilize Gutta-Percha Scraps.*—Scraps of gutta-percha tissue may be made to serve a good purpose by dissolving them in commercial benzole and adding some vermillion or other pigment. When this solution is applied to the necks and stoppers of bottles, a tight-fitting capsule is formed, which is impervious to air, moisture, alcohol and acids, and may be torn off in a moment when desired.—*Druggists Circular.*

---

*The Physiological Action of Aconite.*—In an article in the *Practitioner* Dr. G. H. Mackenzie states that the physiological action of aconite on the respiratory system may thus be summarized:—

1. Its effect on the respiration is primary, and due to the direct action of the drug on the sensory fibres of the vagus, and the respiratory centre.
2. It induces a series of symptoms closely resembling those developed after section of the vagi.
3. It causes death partly by asphyxia, and partly by the variety of collapse spoken of by Brown-Sequard as "characterized by a great diminution of breathing, produced by a peculiar influence on the central organs of respiration, the heart continuing to beat with more or less vigor."—*Med. and Surg. Reporter.*

---

*Action of Chloroform on the Bladder.*—Dr. Reliquet, in his lately published "Lectures on diseases of the Urinary Passages" (Paris, 1878,) states that the complete action of chloroform on the sensibility of the urethra stops at the neck of the bladder, when the latter is the seat of local irritation; in the female, the action of the anæsthetic on the urethra is complete. As for the bladder, the inhalations, far from diminishing its sensibility, appear, on the contrary, to increase it, when it is the seat of some lesion; but, when healthy, the bladder dilates under its influence.—*Med. and Surg. Reporter.*

*Extirpation of Superior Maxilla.*—Dr. Mason presented the left superior maxilla, which he had removed by operation from a female aged thirty-four years, a patient in Roosevelt Hospital. Two years ago she suffered from an abscess in the vicinity of one of the molar teeth. In due time there was a spontaneous discharge of pus. Shortly after this a growth made its appearance in the neighborhood, but it did not increase rapidly, or give rise to any special pain, until two months ago. At the time of her admission to the hospital she was very much run down by excessive pain, which was confined to the left side of the face and to the region of the orbit. The vision of one eye was slightly impaired. The tumor filled the whole of the antrum, projecting eternally into the nares and upward upon the orbit, causing the eye to bulge slightly forward, and also involving the outer surface of the alveolar process and the roof of the mouth. The incision was made by Fergusson's method, and the whole of the superior maxilla removed, leaving its periosteum intact. Tumor was examined by Dr. Delafield, and found to be a spindle-cell sarcoma. He alluded, in connection with this case, to a similar operation which he had performed upon a patient six years ago, with the result of a reproduction of the bone. *Med. Record.*

---

*Malformation of the Skull in Epileptics.*—A writer in the *Annales Psycologiques* states that want of symmetry is nearly always present in the skulls of epileptics. When in the base of the skull it is revealed by a similar condition of the bones of the face, and this condition may easily be detected. In the true epileptic, it consists of a projection of one of the halves of the frontal, generally the right. As a rule, it occupies a position over the eye, but may be found more posteriorly, reaching to the frontoparietal suture. This projection may be detected by sight and touch, and other deformities may then be looked for. These are found in the orbit, the malar bones, and the palate. The soft parts may participate in the deformity, the eyebrows and lips being altered in position, or the folds of the skin more marked on one side than the other.—*Med. and Surg. Reporter.*

---

*Case of Hydrophobia Cured by Oxygen.*—This case is reported by Drs. Schmidt and Zebeden. from Russia. The first symptoms of rabies appeared seventeen days after the injury. The patient was made to inhale three cubic feet of oxygen, and two hours afterwards he was in a state of perfect calm. Two days afterwards the symptoms of rabies reappeared, and another inhalation of oxygen was administered with the same success. This time the inhalation was continued for forty-five minutes. A slight dyspnœa which persisted after the disappearance of the graver symptoms, was treated for three weeks by the monobromide of camphor.—*Lyon Medical.*

*Vegetable Parasites of the Skin.*—Dr. Edward Wigglesworth has studied the characteristics of the vegetable parasites of the skin by a series of inoculations upon himself, giving special attention to the question of their identity or non-identity. The following are the results he has obtained: 1. All vegetable parasites of the skin are not inoculable at all times and upon all persons. II. Varying degrees of intensity, or duration of application, are needed for successful inoculation of different parasites upon the same skin; the severer cases requiring more thorough inoculation. III. A healthy skin may resist the action of the less severe but more widely-spread mycoses but, yields to the more thorough inoculation of the more severe and rare forms, showing that the resistant power of the soil furnished is a factor to be regarded. IV. Extension and intension are in inverse ratio to each other. The milder mycoses are the more common, and point to an origin upon skins below par in vigor. V. The various mycoses of the human integument possess each its own distinguishing characteristics, although a transitory stage of growth of one of them may, in rare cases, as in the "ringworm stage" of favus, simulate in appearance one of the forms, temporary or more permanent, of an apparently different species. VI. While botanical and clinical observations are so at variance in reference to the identity or non-identity of the mycoses, this question must be regarded as still undecided.—*Archives of Dermatology.*

---

*Lead-Poisoning by Flour.*—Dr. Alford details the particulars of a local outbreak of lead-poisoning, which occurred in one part of the Taunton Rural Sanitary District, but in houses far apart. He analyzed the water, cider, preserved fruits, etc., but without detecting any lead. On careful inquiry, however, he found that the families affected had one thing in common, viz.: they all had their flour from the same mill. An analysis of this flour showed the presence of lead, and an examination of the mill revealed the fact that the holes in the stones had been filled up with lead. The lead was removed, and the disease gradually disappeared. Some of the cases were of a very severe form. It appears, on further inquiry, that it is by no means an uncommon thing for the holes in mill-stones to be filled with lead, a fact which is worth remembering.—*Med. Record.*

---

*Antidote to Carbolic Acid.*—On the recommendation of Prof. Baumann, Dr. Sanftleben used sulphuric acid in several cases of poisoning by carbolic acid with the best success, the phenol combining with the acid to form phenyl-sulphuric acid, which is not poisonous. He administered it in a mixture composed of diluted sulphuric acid 10:0, mucilage of gum 200:0, and simple syrup 30:0 grammes, in doses of a tablespoonful every hour.—*Pharm. Ztg. f. Russl.*

*Hypodermic Injections of Chloroform.*—These injections have been highly recommended by eminent physicians as a substitute for morphine. It is claimed that they are painless, that they relieve pain rapidly and for several hours, and that they are entirely innocuous, being followed by neither local nor general symptoms. Dr. Jochheim, of Darmstadt, however, reports a case in which the injection of only ten drops of chloroform, which is only half what is frequently administered, was followed by severe local disturbances. Five hours after the injection a violent local inflammation set in, and in twenty-four hours a hard, black slough had formed, which was not cast off by suppuration until six weeks afterwards.—*Allg. Med. Cent. Zeit.*

---

*Chloroform Narcosis Cured by Nitrite of Amyl.*—An English Journal reports a case of threatened death from chloroform, in which the patient was resuscitated by the inhalation of a few drops of nitrite of amyl. The indication for the use of amyl is furnished by the sudden failure of the pulse.

Another means of treating a threatening narcosis caused by chloroform is recommended by M. Baillee, of Belgium. It consists in the introduction of a small piece of ice into the rectum. A moderate degree of pressure suffices to overcome the resistance of the sphincter. The ice melts in the intestine, and immediately excites a deep inspiration, which is followed by the reestablishment of natural respiration and of the cardiac functions.

This treatment might also prove serviceable to excite respiration in the new-born child.—*Med. Record.*

---

*Disdvantage of Salicylic Acid as a Dentifrice.*—Dr. Dobrowski reports, in the *All. Med. Central-Zeit.*, an unpleasant odour to the breath after long use of salicylic acid, either in mouth washes, or as a tooth powder. If salicylic acid remains in the mouth, it decomposes in very small quantity the sulpho-cyanide of potash, one of the constituents of the saliva whose function is to arrest decomposition in food debris. It is the odour of decomposition thus permitted which is communicated to the breath. —*The Doctor.*

